# Retrieval Practice Improves Recollection-Based Memory Over a Seven-Day Period in Younger and Older Adults

**DOI:** 10.3389/fpsyg.2019.02997

**Published:** 2020-01-22

**Authors:** Catherine-Noémie Alexandrina Guran, Jovana Lehmann-Grube, Nico Bunzeck

**Affiliations:** Institute of Psychology I, University of Lübeck, Lübeck, Germany

**Keywords:** retrieval practice, testing effect, aging, recollection and familiarity, temporal dynamics

## Abstract

Retrieving information improves subsequent memory performance more strongly than restudying. However, despite recent evidence for this retrieval practice effect (RPE), the temporal dynamics, age-related changes, and their possible interactions remain unclear. Therefore, we tested 45 young (18–30 years) and 41 older (50 + years) participants with a previously established RP paradigm. Specifically, subjects retrieved and restudied scene images on Day 1; subsequently, their recognition memory for the presented items was tested on the same day of learning and 7 days later using a remember/know paradigm. As main findings we can show that both young and older adults benefited from RP, however, the older participants benefited to a lesser extent. Importantly, the RPE was present immediately after learning on Day 1 and 7 days later, with no significant differences between time points. Finally, RP improved recollection rates more strongly than familiarity rates, independent of age and retrieval interval. Together, our results provide evidence that the RPE is reduced but still existing in older adults, it is stable over a period of seven days and relies more strongly on hippocampus-based recollection.

## Introduction

Repeated encoding is a common way of learning new materials, however, retrieving new material (i.e., attempting to recall material) is more effective (Karpicke et al., 2009). The benefit of retrieval practice (RP) is stronger even than elaborative encoding methods (Karpicke and Blunt, 2011), and this “retrieval practice effect” (RPE, or “testing effect”) is present across different stimulus modalities (Abbott, 1909; Spitzer, 1939; Karpicke and Roediger, 2008; Karpicke and Blunt, 2011). In terms of temporal dynamics, the RPE is considered to be stable across retention intervals (i.e., the interval between RP and final recall) of days, and weeks (Butler and Roediger, 2007; Bouwmeester and Verkoeijen, 2011). However, the literature diverges on whether it is present immediately after RP (Roediger and Karpicke, 2006; Toppino and Cohen, 2009), and whether its strength decreases, increases or remains unchanged with longer retention intervals. One possible explanation for these discrepancies is that previous studies used different designs, for instance, regarding how retrieval was practiced (e.g., free recall vs. recognition), and the nature of subsequent memory tests. Apart from open questions regarding the temporal stability of the RPE, possible age-related changes, as well as the underlying mechanisms, also remain unclear. Addressing these issues was the focus here.

In view of its pervasive long-lasting memory effects, RP is a potential candidate to improve memory not only in young but also older adults. This is particularly important since older age is typically associated with a decline in declarative memory functions, starting as young as 50 years of age (Crook et al., 1986). At the neural level, these declines have been linked to specific brain structures (Hedden and Gabrieli, 2004). Particularly, the medial temporal lobe (MTL), including the hippocampus and surrounding cortex which plays a key role in recognition memory, declines with age, starting – on average – in the middle of the sixth life decade, with considerable inter-individual variation (Raz et al., 2005). Recognition memory is associated with the MTL (Diana et al., 2007), and is often investigated using the remember/know paradigm (Tulving, 1985), which assumes that recognition can either be associated with specific details or associations of the encoding episode (i.e., recollection) or take place in the absence of such associative-recollective experience (i.e., familiarity). Support for this dual-process idea (Yonelinas et al., 1996, 2010) comes from functional imaging studies that suggest different regions of the MTL to be involved in the two different memory experiences.

A few recent studies indicate that the RPE may be impaired with age: while RP has been shown to still provide memory benefits in older adults (Meyer and Logan, 2013), the benefit is smaller than in young adults (Guran et al., 2019), and sometimes dependents on other characteristics of the task such as feedback (Tse et al., 2010). Thus, in general, the effect appears less robust in older than in young adults, and age-related neurobiological changes might play a role in explaining the behavioral differences in RP between age groups. However, it still remains unclear whether, in the context of RP, age affects specific forms of recognition memory (i.e., recollection vs. familiarity), and whether the effects show the same temporal profiles as in young participants.

A range of (mechanistic) theories has been proposed to explain the RPE. The Episodic Context Account (Karpicke et al., 2014) suggests that the RPE is associated with recreating the initial learning context, updating the context through retrieval, and updating the search set based on successful retrieval. However, this approach struggles to account for the findings of RPEs for novel stimuli: RPEs have been shown to extend even to completely novel stimuli that are shown in a retrieval context (Chan et al., 2006; Cho et al., 2017; Guran et al., 2019). If the RPE relies on a recreation of the learning context, no benefit would be expected for novel stimuli, which have never been encountered. Alternatively, Antony et al. (2017) proposed that RP acts as a fast-route to consolidation, whereby RP leads to memory traces that are potentially less hippocampus-dependent. Therefore, both theories make different predictions regarding the effects of RP on hippocampus-dependent memories. However, empirical evidence remains scarce since only a few studies have investigated the RPE in a source recognition memory paradigm (or investigated the RPE in the MR scanner). Existing studies indicate specific effects of RP on recollection but not familiarity ratings (Chan and McDermott, 2007), which would be in line with the Episodic Context Account Theory. Indeed, Verkoeijen et al. (2011) could replicate this observation and reported that the specific effects of RP on recollection were even more pronounced when feedback or cues were presented during encoding. However, the temporal stability of recognition-specific RPE and possible age-related changes remain unclear.

The personality trait “Novelty Seeking,” which is associated with exploratory activity in response to novelty, has been linked with the brain’s dopaminergic system (Benjamin et al., 1996; Ebstein et al., 1996), including the substantia nigra/ventral tegmental area (SN/VTA) (Krebs et al., 2009). This is an interesting observation since dopamine also plays an important role in memory functions (Wittmann et al., 2005; Schott et al., 2006; Chowdhury et al., 2012), and its bioavailability is known to decrease in old age, especially in the SN/VTA due to transporter and receptor loss (Fearnley and Lees, 1991; Bäckman et al., 2006). Under the assumption that Novelty Seeking is an indirect measure of the brain’s dopaminergic system, then individuals with higher Novelty Seeking traits might, at the same time, show a better functioning memory, as well as larger increases in memory due to RP.

To further investigate the RPE, especially regarding temporal stability and possible age-related changes, a recollection/familiarity approach (Tulving, 1985; Yonelinas, 2002) was used. Specifically, a group of older and younger participants were presented with a series of scene images both in an encoding and retrieval context. Their recognition memory for the presented images was tested on the same day, and 1 week after the initial learning phases using a remember/know paradigm. We expected an RPE in both young and older adults (hypothesis 1), which should be reduced in the latter (hypothesis 2). The central aim of this study was to investigate the roles of recollection and familiarity in the RPE, and we expected a remember-specific RPE (hypothesis 3). Due to our previous work (Herweg et al., 2018; Guran et al., 2019), we also expected the RPE to be present immediately after RP and a week later (hypothesis 4), irrespective of stimulus Novelty (Cho et al., 2017; Guran et al., 2019) (hypothesis 5). In addition, we explored a possible relationship between Novelty Seeking and the RPE (hypothesis 6).

## Materials and Methods

### Participants and Sampling

Participants were recruited online using the Online Recruitment System for Economic Experiments (ORSEE, Greiner, 2015). All participants were right-handed, spoke and understood German fluently, and had no personal history of neurological or psychiatric disorders. Older subjects (above 50 years of age) were additionally screened for cognitive impairment with the Montreal Cognitive Assessment Scale (MoCA, Nasreddine et al., 2005), and excluded if they scored lower than 22 points, which is considered a threshold for mild cognitive impairment (Freitas et al., 2013). A total of 49 young participants, and 46 older participants were measured. From this initial sample, nine participants had to be excluded due to a below-threshold MoCA score (one), later reported brain hemorrhages (one), technical difficulties (two), no show to the follow-up measurement (two), falling asleep during measurement (one), or not following task instructions (two). Demographic information for the remaining sample can be found in Table 1.

**TABLE 1:** Demographic data for the younger and older subsample.

	**Age**		**Sex**		**MoCA**		**Total**
**Age group**	**Mean ± SD**	**Range**	**♀**	**♂**	**Mean ± SD**	**Range**	
Young	24.18 ± 3.66	18–33	24	21	–		45
Older	67.61 ± 8.3	50–82	19	22	26.6 ± 1.8	23–30	41
Total			42	44			

### Experimental Design

**FIGURE 1:**
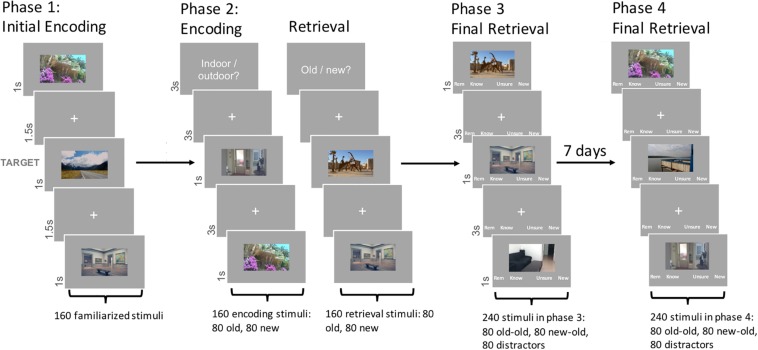
Experimental paradigm. Phase 1 familiarizes participants with 160 stimuli through a target detection task. Stimuli first shown in Phase 1 (old stimuli) are then shown in Phase 2, interspersed with 160 new stimuli, in either one of two tasks, (a) the Encoding Task (ENC), in which images are categorized into indoor or outdoor, or (b) the Retrieval Task (RET), in which participants have to determine whether they have seen the stimulus already or not. In Phase 3, half of all previously encountered stimuli are shown with 80 new distractors and participants have to recall the stimuli, giving recollection/familiarity responses in form of “remember” or “know” responses. Phase 4 follows the same rationale as Phase 3, with the other half of the stimuli, and a week later.

The experimental paradigm was based on Herweg et al., 2018. The procedure consisted of four phases (see Figure 1). In Phase 1, participants were familiarized with 160 outdoor and indoor images (80 each) by means of a target detection task: The target stimuli (one indoor and one outdoor picture) were presented initially for 12s. Subsequently, 160 images for familiarization were presented three times each, for 1s in pseudorandom order intermixed with 9% of target trials (i.e., 48 target and 480 non-target trials). Each image was followed by an inter-stimulus interval of 1.5s (white fixation cross on gray background). Participants had 2s to respond to the target stimuli with a button press, and had the opportunity to pause every 96 trials. Target stimuli were not shown again outside of Phase 1.

In Phase 2, participants had to perform two different, randomly alternating tasks, while viewing 160 new stimuli randomly intermixed with the 160 familiarized stimuli. The tasks were designed to induce an encoding and a retrieval context for half of the new and familiarized stimuli each. In the encoding task (ENC), participants gave simple indoor/outdoor categorization judgments, by means of button presses. In the retrieval task (RET), participants gave an old/new recognition judgment, again through button presses. The combination of the two factors Task (ENC/RET) and stimulus Novelty (old/new) resulted in a 2 × 2 repeated measures design with 80 stimuli per condition. Task blocks were eight trials long, each block containing four old and four new stimuli in random order. An instruction screen informed participants about the upcoming task prior to the start of each block. Images were presented for 1s with an inter-stimulus interval of 3s (fixation cross). Participants gave their response within 2.8s using their right index and middle finger. Response-button mappings were counterbalanced across participants. Participants could make a self-paced pause every 64 trials.

In Phase 3, participants performed a surprise recognition task 5–10 min after the end of the second phase. One hundred sixty previously encountered stimuli, counterbalanced for location (indoor/outdoor), stimulus Novelty and Task in Phase 2, were intermixed with 80 unseen distractor images (i.e., 80 stimuli from the encoding context, 80 stimuli from the retrieval context and 80 unseen distractors). Each image was presented for 1s with four response options in German below the image: Remember – Know – Unsure – New (or in opposite order, depending on key mapping in Phase 2). Participants were instructed, orally and in writing, about the meaning of each response option. They were asked to choose “Remember” when they recognized a picture *and* could recollect specific thoughts or associations linked to the study episode (Recollection). They were asked to choose “Know” when they recognized the picture but were not able to recall specific associations related to the study episode (Familiarity). “Unsure” was to be pressed when they did not know whether a picture was old or new, and “New” when they were sure they had not seen the picture before. The inter-stimulus interval was 3s (fixation cross and response options) during which participants could still give their response using the computer keyboard. Participants could make a self-paced pause every 60 trials. Phases 1, 2, and 3 were all performed consecutively, with small breaks (5–10 min) between them.

The task in Phase 4 was identical to Phase 3. However, it was conducted a week (7 days) later. Participants were shown the remaining 160 stimuli from Phase 2 that had not been presented in Phase 3, as well as another 80 previously unseen (distractor) stimuli (i.e., 80 stimuli from the encoding context, 80 stimuli from the retrieval context and 80 unseen distractors).

Images were randomly assigned to the different phases and conditions for each subject. To control for effects of illumination, mean luminance on each color channel (R,G,B) was set to 127 (scale from 0 to 288) and images were presented on a gray background of equal luminance. Prior to each phase of the experiment, participants completed a brief training session. Images used during the training phase were different from those used during testing. After completing Phase 4 of the experiment, participants filled out a computerized and German version of the Temperament and Character Inventory (TCI, Cloninger, 1987). A further 55 TCI datasets of participants were available and included in the TCI analysis. These data came from an experimental paradigm that was identical except for the response modality in Phases 3 and 4 (confidence ratings instead of recollection/familiarity) as well as the retention interval (one instead of 7 days, Guran et al., 2019).

### Statistical Analyses of Behavioral Data

Accuracy of behavioral responses was assessed on the basis of signal detection theory (Stanislaw and Todorov, 1999). For target detection during Phase 1, hits were defined as correctly detected targets. For old/new categorization during Phase 2, hits were defined as old stimuli correctly classified as old. For indoor/outdoor categorization during Phase 2, hits were defined as indoor images correctly classified as indoor. For Phase 3 and 4, hits were defined separately for old stimuli that were remembered, and old stimuli that were recognized, but not remembered (known). Specifically, d′ (d-prime) was calculated by subtracting the inverse phi (conversion of probabilities into *z*-scores according to the normal cumulative distribution function) of the hit rate from the inverse phi of the false alarm rate for each subject and condition. As the inverse phi of 0 and 1 is −∞ and ∞, respectively, 0.5 was added to the number of hits and false alarms and 1 was added to the number of signal and no signal trials (Stanislaw and Todorov, 1999).

Data were statistically analyzed using a 2 (Memory: remember/know) × 2 (Retrieval Day: Day 1/Day 7) × 2 (Task: ENC/RET) × 2 (stimulus Novelty: old/new) × 2 (Age: young/older) repeated measures ANOVA, frequentist as well as Bayesian *T*-Tests, Kolmogorov-Smirnov Tests, and Levene-Tests where appropriate and necessary. Alpha-levels were Bonferroni corrected for multiple comparisons, when appropriate. To address the problem of overfitting we also conducted a separate reduced ANOVA (without factors Day and stimulus Novelty).

The TCI dimension “Novelty Seeking,” as well as its individual subscales, was used to calculate Spearman correlations with behavioral benefit from RP. The individual RP benefits were calculated by subtracting d′_Retrieval_ – d′_Encoding_ for each participant, thus positive values indicating higher memory performance in Retrieval as compared to Encoding. Retrieval practice benefits were calculated separately for Phase 3 and 4.

Calculation of d′ and hitrates was performed in MATLAB 2019a (The MathWorks; RRID:SCR_001622), whilst the analysis was performed with IBM SPSS statistics, version 25, as well as JASP (JASP Team, 2019). Barplots were made using Gramm (Morel, 2018).

## Results

### Phase 1

Participants’ accuracy in the first phase, which consisted of a target detection task, was similarly high in both age groups, *d*′*_young_*(M ± SD) = 5.36 ± 0.33, *d*′*_elderly_* = 5.53 ± 0.23. As the assumption of homogeneity of variance was violated, both for the mean (Levene-Test: *F*_Mean_ = 10.93, *p* < 0.01) and the median statistic (*F*_Median_ = 6.84, *p* < 0.05), an independent-samples *T*-Test with bias-corrected bootstrapping (1000 samples) was performed. The results suggest a higher performance in the older age group (*T*_84_ = −2.62, *p*_bootstrapped_ = 0.008).

### Phase 2

In Phase 2, data were analyzed in a 2 × 2 repeated measures ANOVA, with Task (ENC/Ret) as a within-subjects factor, and age group as a between-subjects factor. Data residuals were normally distributed except d′_Encoding_ in the older sample, *p* < 0.001 (Kolmogorov–Smirnov Test: *D*_41_ = 0.23), and variance was homogenous (lowest *p* > 0.36). There was a main effect of task for response accuracy d′ (Task: *F*_1_,_84_ = 256.53, *p* < 0.001, η_*p*_^2^ = 0.753); a *post hoc* paired *T*-test revealed that participants responded more accurately in the encoding than the RET (*T*_85_ = 16.13, *p* < 0.001). There was no main effect of Age group, and no significant interaction of Task and Age group (*p* > 0.8). Since d′ for the retrieval condition was calculated based on responses for old and new images, these data cannot be analyzed in a 2 × 2 × 2 repeated measures ANOVA.

Furthermore, we calculated a 2 × 2 ANOVA based on the reaction times for each Task and each Age group. There was a main effect of Task (*F*_1_,_84_ = 245.21, *p* < 0.001, η_*p*_^2^ = 0.745), and of Age group (*F*_1_,_84_ = 12.74, *p* < 0.01, η_*p*_^2^ = 0.98). There was no interaction (*p* > 0.19). While homogeneity of variance was present (smallest *p*-value = 0.2), data residuals were non-normally distributed for the young participants (*p* < 0.05), thus we used bootstrapped *T*-Tests. Both younger and older participants were faster in the Encoding as compared to the Retrieval task (young: *T*_44_ = −9.7, *p*_bootstrapped_ < 0.01, older: *T*_40_ = −12.77, *p*_bootstrapped_ < 0.01; α = 0.025). Younger participants were faster than older participants in both Task conditions (Encoding: *T*_84_ = −3.23, *p*_bootstrapped_ < 0.01, Retrieval: *T*_84_ = −3.58, *p*_bootstrapped_ < 0.01; α = 0.025). The means and SDs of reaction times can be found in Table 2.

**TABLE 2:** Means and standard deviations of reaction times in the different tasks in Phase 2.

			**Mean (msec)**	**SD (msec)**
Age group	Young	Encoding	793	123
		Retrieval	924	153
	Older	Encoding	887	147
		Retrieval	1043	153

### Phases 3 and 4

Addressing our hypotheses 1–5, Phases 3 and 4 were analyzed together as part of one 2 × 2 × 2 × 2 × 2 repeated measures ANOVA, with Memory (remember/know), Phase (Day 1/Day 7), Task (ENC/RET), stimulus Novelty (old/new stimuli) as within-subjects factors, and age group (young/older) as between-subjects factor. Homogeneity of variance was violated in only three of the 16 variables used for the ANOVA, smallest *p* = 0.034 (Levene-Test). Normality of residuals was violated in six out of the 32 variables (16 split by age group), smallest *p* = 0.001 (Kolmogorov–Smirnov). However, as the sample size was large (*n* > 40 per age group) and there do not seem to be suitable non-parametric analogs of a mixed 2 × 2 × 2 × 2 × 2 ANOVA, we conducted the ANOVA as planned. We corrected for multiple tests for the effects of interest (see hypotheses 1–5). The Bonferroni-adjusted α-level for main effects and interactions of interest in this ANOVA was *p* < 0.01. For all other effects, results were exploratory, although when correcting for all tests conducted by the 5-way ANOVA (31 in total), almost all remained significant nonetheless (α = 0.0016).

#### Main Effects

**FIGURE 2:**
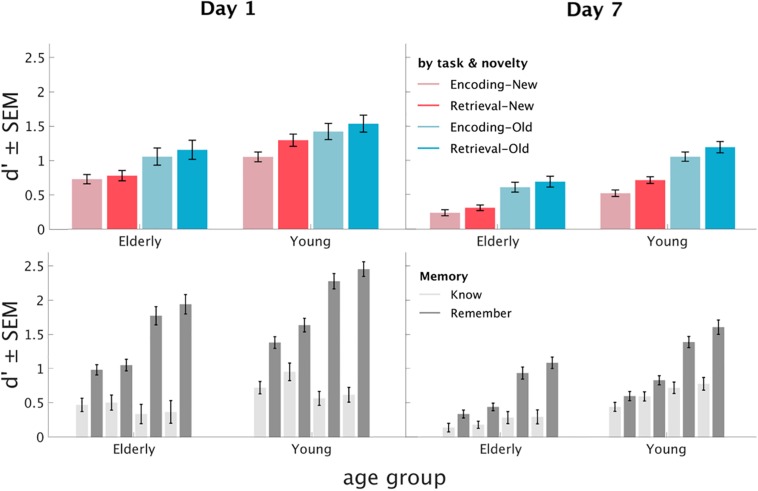
Memory performance for Day 1 and Day 7. *D*′ values for all conditions: Day (separate plots), Age group (*x*-axis), Memory (lightness), Task (color saturation), stimulus Novelty (color temperature, blue = old).

Regarding our hypothesis 1 of increased memory accuracy in the Retrieval Task, there was a main effect of Task (*F*_1_,_84_ = 101.77, *p* < 0.001, η^2^ = 0.55), as well as main effects of Memory (*F*_1_,_84_ = 89.24, *p* < 0.001, η^2^ = 0.52), Day (*F*_1_,_84_ = 208.3, *p* < 0.001, η^2^ = 0.71), stimulus Novelty (*F*_1_,_84_ = 360.54, *p* < 0.001, η^2^ = 0.81), and Age group (*F*_1_,_84_ = 34.51, *p* < 0.001, η^2^ = 0.291). As can be seen in Figure 2, stimuli that had been presented in the RET in Phase 2 were remembered significantly better than stimuli presented in the ENC (*T*_85_ = −11.18, *p* < 0.001). Furthermore, participants showed higher remember than know rates (*T*_85_ = 9.52, *p* < 0.001), higher memory accuracy on the first day in comparison to the recall 7 days later (*T*_85_ = 22.42, *p* < 0.001), and higher memory accuracy for older stimuli, i.e., those that were shown initially in Phase 1 (*T*_85_ = 21.29, *p* < 0.001). Younger participants had higher scores as compared to older participants (*T*_84_ = 3.57, *p* < 0.01).

#### Interactions

In total, there were five significant 2-way interactions, and three significant 3-way interactions. Figures are provided for interactions that were related to the hypotheses. *T*-Tests were performed to disentangle the two-way interactions, as well as for the three-way interaction, which related to one of the hypotheses.

##### Two-Way Interactions

**FIGURE 3:**
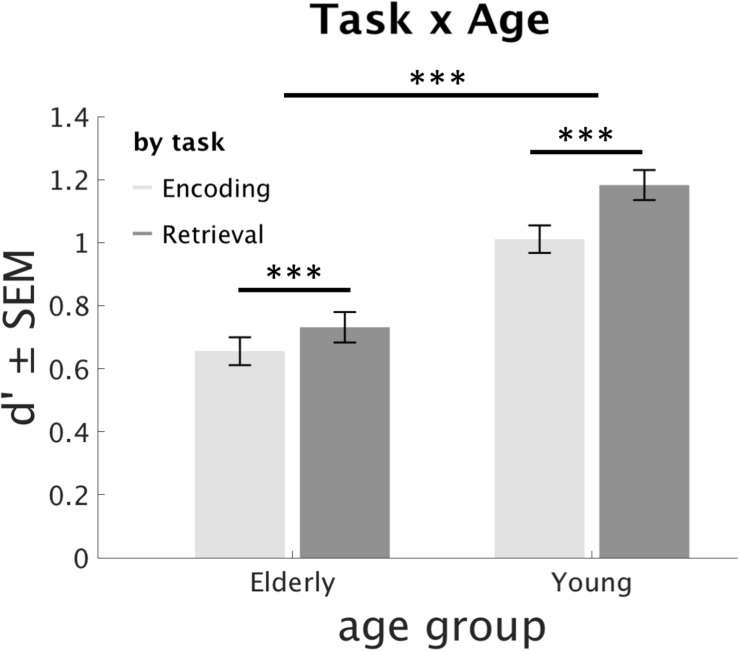
Differential effect of retrieval practice (RP) on age group. Both young and older participants show the RPE, but it is significantly larger in the young. ^∗∗∗^*p* < 0.001.

Regarding our hypothesis 2 of an age-related decrease of the RPE, there was a significant interaction of Task × Age (*F*_1_,_84_ = 15.26, *p* < 0.001, η^2^ = 0.15). Separate paired *T*-Tests (averaged across stimulus Novelty, Memory and Day) showed that both young and older participants benefit from RP (young: *T*_44_ = 10.94, *p* < 0.001, older: *T*_40_ = 6.19, *p* < 0.001) but that the RPE was larger for young participants than older ones (independent samples *T*_84_ = 4.7, *p* < 0.001), see Figure 3.

**FIGURE 4:**
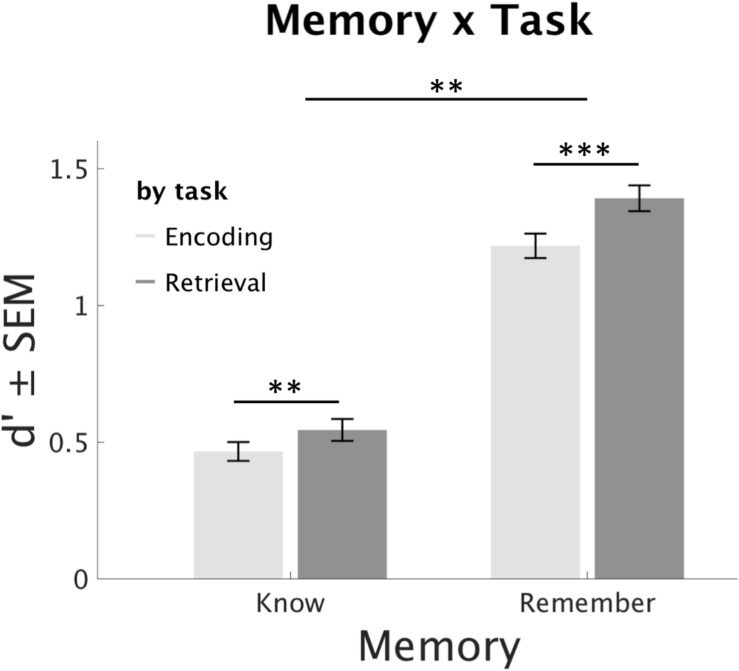
Effects of retrieval practice on type of memory. Retrieval increases both types of recognition memory, but this effect is more pronounced for remember rates. ^∗∗∗^*p* < 0.001, ^∗∗^ *p* < 0.01.

Regarding our hypothesis 3, addressing differential influences of recollection and familiarity within the RPE, there was a significant interaction of Memory × Task (*F*_1_,_84_ = 8.21, *p* = 0.005, η^2^ = 0.09). Dependent samples *T*-Tests (averaged across stimulus Novelty, Age and Day) showed higher remember and know responses in the RET as compared to the ENC Task (know: *T*_85_ = 3.57, *p* < 0.01, remember: *T*_85_ = 8.54, *p* < 0.001). Importantly, the influence of RP was more pronounced for remember responses (*T*_85_ = 2.88, *p* = 0.005), see Figure 4.

There was a significant interaction of Memory × Retrieval Day (*F*_1_,_84_ = 43.65, *p* < 0.001, η^2^ = 0.34). Dependent samples *T*-Tests (averaged across stimulus Novelty, Age and Task) revealed that remember responses were higher on Day 1 as compared to Day 7 (know: *T*_85_ = 2.29, *p* = 0.024, n.s., remember: *T*_85_ = 13.62, *p* < 0.001). The difference from Day 1 to Day 7 was stronger for remember than know responses (*T*_85_ = −6.658, *p* < 0.001).

There was an interaction of Day × stimulus Novelty (*F*_1_,_84_ = 17.28, *p* < 0.001, η^2^ = 0.17). Dependent samples *T*-Tests (averaged across Memory, Age and Task) revealed that while both old and new stimuli were remembered less on Day 7 as compared to Day 1 (old: *T*_85_ = 20.07, *p* < 0.001, and new: *T*_85_ = 18.92, *p* < 0.001), there was a trend for this effect to be slightly more pronounced for new as compared to old stimuli (*T*_85_ = 1.73, *p* = 0.088).

Finally, there was a significant interaction of Memory × stimulus Novelty (*F*_1_,_84_ = 128,27, *p* < 0.001, η^2^ = 0.6). Dependent samples *T*-Tests (averaged across Day, Age and Task) showed no significant difference in know responses for old vs. new stimuli (*p* > 0.9). However, remember responses for old stimuli were significantly higher as compared to new stimuli (*T*_85_ = 11.41, *p* < 0.001). The corrected α-level for the *post hoc T*-tests following significant two-way interactions was α = 0.0167.

There were no other significant two-way interactions, including Task × stimulus Novelty (*p* > 0.15) or Task × Day (*p* > 0.7, hypothesis 4). To further investigate the absent effect of Task × Day, we conducted a Bayesian paired-samples *T*-Test to ascertain whether the retrieval benefit was different on Day 1 vs. Day 7. Compatible with the ANOVA results, there was only anecdotal evidence for a difference between the two timepoints (BF_10_ = 2.38). The retrieval benefit was insignificantly smaller on Day 7 (Day 1: *d*′_*Diff*_ = 0.3; Day 7: *d*′_Diff_ = 0.2).

##### Three-Way Interactions

**FIGURE 5:**
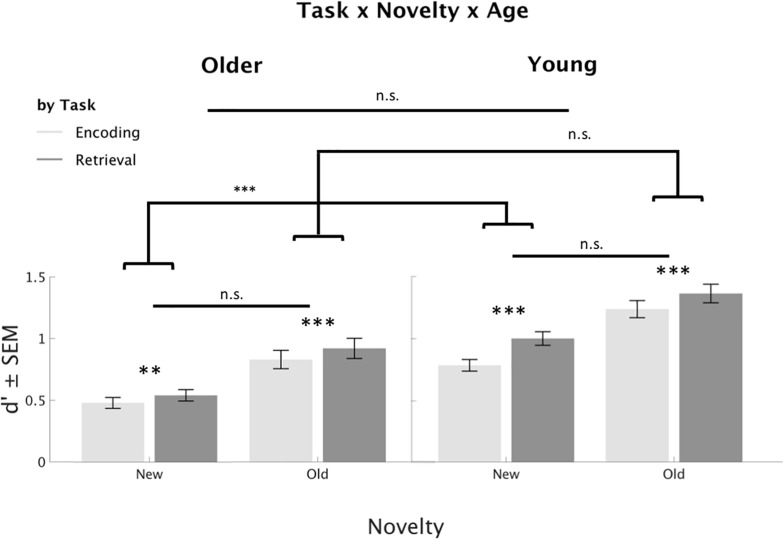
Interaction of Task, stimulus Novelty, and Age group. The retrieval practice effect was significant for old and new stimuli, and there was no significant difference in strength between old and new stimuli. However, the difference between the RP for new stimuli seemed more pronounced in young adults in comparison to older participants. ^∗∗∗^*p* < 0.001; ^∗∗^*p* < 0.0056.

Related to our hypothesis 5, namely that the RPE would be present for old and new items, there was a trend-level interaction (corrected α-Level = 0.0016) between Task, stimulus Novelty, and Age (*F*_1_,_84_ = 7.21, *p* = 0.009, η^2^ = 0.08), see Figure 5. *Post hoc T*-Tests (averaged across Memory and Day) revealed significant RPEs for old and new stimuli for both young and older participants (see Figure 5). The interaction was driven by a larger difference between RPEs in new, but not old, stimuli in the young adults as compared to older adults. Note, however, that the direct comparison of RPEs for Old and New stimuli within each age group did not reach significance (*p* > 0.2). The Bonferroni corrected alpha-level for *post hoc T*-Tests following significant interactions was α = 0.0056.

There was a significant interaction between Day, stimulus Novelty, and Age (*F*_1_,_84_ = 10.81, *p* = 0.001, η^2^ = 0.11), and a significant interaction of Memory, Day, and stimulus Novelty, *F*_1_,_84_ = 35.82, *p* < 0.001, η^2^ = 0.3. Since these two interactions were not part of our hypotheses and since they did not include Task, they were not followed up by *post hoc T*-tests. No other three-, four-, or five-way interactions were found.

### TCI-Results

Addressing hypothesis 6 on a link between trait Novelty Seeking and the RPE, we calculated Spearman correlations between RP benefit across days, as well as for each day separately, and the TCI “Novelty Seeking” scale – including its subscales (Exploratory Excitability, Impulsiveness, Extravagance, Disorderliness) – in a large sample comprising data from this and a previously reported study (Guran et al., 2019, see methods). There were no significant correlations (*N* = 141, Bonferroni corrected alpha-level: α = 0.003). However, a trend for a correlation between the RPEs, averaged across both days (*r* = 0.218, *p* < 0.009), on Day 1 (*r* = 0.181, *p* = 0.032), and on Day 7 (*r* = 0.165, *p* = 0.05), with the TCI’s “Novelty Seeking” subscale “Exploratory Excitability” was found (see Table 3). Furthermore, there was a trend for a correlation between the RP benefit overall and the subscale “Impulsivity” (*r* = −0.166, *p* = 0.049).

**TABLE 3:** Spearman correlation between TCI novelty seeking and RPEs, *N* = 141.

			**TCI novelty seeking subscales**				**Novelty seeking**
			**Exploratory excitability**	**Impulsiveness**	**Extravagance**	**Disorderliness**	**(includes all 4 subscales)**
RPEs	Overall	*r*	0.218*	–0.166	0.01	0.125	0.038
		*p*	0.009	0.049	0.91	0.14	0.65
	Day 1	*r*	0.181*	–0.118	–0.03	0.16	0.052
		*p*	0.032	0.17	0.73	0.07	0.54
	Day 7	*r*	0.165	–0.128	–0.002	–0.009	–0.004
		*p*	0.050	0.131	0.98	0.92	0.96

*p < 0.05. No significant effects after correction for multiple comparisons (α = 0.003).

### Reduced Models and Exploratory Analyses

In order to avoid the possibility of false positives as a result of overfitting, we conducted a simpler 2 × 2 × 2 ANOVA with the factors Memory, Task, and Age, excluding the factors Day and stimulus Novelty. It also revealed significant main effects of Memory, Task, and Age (all *p*-values < 0.001), as well as interactions of Task × Age, and Memory × Task (both *p*-values < 0.01). No other interactions were significant.

Furthermore, since we investigated a broad range of older participants (50–82 years), we separated this group in young-old (50–65, *n* = 15) and old-old participants (60–82, *n* = 26). However, a 2 × 2 × 2 ANOVA with Memory, Task, and old-age subgroups as a factor did not reveal a main effect of Age (*p* = 0.1). A Bayesian two-sample *T*-Test revealed that there was moderate evidence for the groups to be the same, BF_01_ = 3.1.

## Discussion

Retrieval practice reliably improves long-term memory, but its temporal stability, relation to recollection/familiarity, aging, and Novelty Seeking has not been systematically investigated. In this study, we could show that the RPE remained stable across a 7-day period, in young as well as older adults, while older adults benefit less from RP. The RPE was present for both previously encountered (old) and novel (new) stimuli, which suggests that enhanced difficulty (or effort) may play a role. Importantly, RP improved both types of memory rates, but the effect was more pronounced for recollection as compared to familiarity indicating a differential effect of RP on recognition memory. Lastly, no convincing evidence was found in favor of a relationship between Novelty Seeking and RP benefits.

As hypothesized (hypothesis 1), RP improved long-term memory in both young and older subjects (Figure 3), which is in line with evidence that RP or testing leads to better memory outcomes compared to restudy or elaborative encoding strategies (Karpicke and Blunt, 2011; Meyer and Logan, 2013; Rowland, 2014; Guran et al., 2019). The RPE has been present in many different settings (word lists, scientific texts, and now pictures) and seems to be quite general. However, whether our findings (age and time) generalize to other stimulus material, or other memory modalities, remains to be studied. In any case, RP seems to be an effective way of improving memory even in clinical populations, such as patients with traumatic brain injury (Sumowski et al., 2014) or multiple sclerosis (Sumowski et al., 2010). In contrast, the effectiveness of RP in other disorders that directly affect dopamine levels in the brain, such as Parkinson’s disease, remains unclear.

In terms of age-related modulation of the testing effect, there is no clear consensus in the literature whether RP reliably leads to improvement in older adults or not, mainly due to a sparsity in studies investigating age in a comparative setting. Some studies find similar improvements in young and older adults (Balota et al., 1989; Meyer and Logan, 2013) while others report a reduction (Guran et al., 2019) or decreased robustness (Tse et al., 2010), of the RPE in older adults. One possibility is that differences in stimulus materials (e.g., words vs. pictures), and paradigms (free vs. cued recall) might explain the differing findings, which at a more general level, reduces comparability between studies. Moreover, aging is associated with increased interindividual differences regarding a variety of cognitive abilities (e.g., Hedden and Gabrieli, 2004), which may have also differed between studies. Although our data do not allow a clear explanation of different findings, it adds evidence in favor of the notions that (a) older adults also benefit from the RP, and (b) this benefit may be reduced in strength (hypothesis 2). The reduced benefit for RP for older adults is particularly notable, considering that in Phase 1 older adults performed slightly better, which suggests they were more attentive to the task.

As hypothesized and previously shown, the RPE was present immediately and 7 days later (hypothesis 4), showing that RP-related memory improvement is fast and stable. This conclusion is in line with the “Fast Route to Consolidation” hypothesis, suggesting that RP leads to fast consolidation by enhancing the integration of new information into preexisting neocortical networks (Antony et al., 2017). Specifically, this mechanism supposedly relies on fast modifications of neocortical representations, which have been shown elsewhere (Tse et al., 2007; Brodt et al., 2018). Importantly, the RPE was not only fast, but also stable over 7 days, suggesting that RP leads to a long-term modification of memory contents, which is in line with previous studies (Butler and Roediger, 2007; Bouwmeester and Verkoeijen, 2011). However, other studies (Roediger and Karpicke, 2006; van den Broek et al., 2014) have not found RPEs in response accuracy immediately after restudy/retrieval sessions. This discrepancy between the above mentioned literature and our findings here and elsewhere (Guran et al., 2019) could be due to the stimulus material and response mode: in previous studies, word pairs were learned and actively recalled, while in our study, we used scene stimuli that were shown again to assess memory. The differences between active recall and recognition memory and how they affect the RPE should be compared directly to see whether the different results possibly reflect different underlying processes within the RP paradigm. Investigating the exact neural processes involved, for instance with fMRI or EEG, would be an important step to further understand the RPE. Interestingly, there was no significant interaction of Day × Age or Day × Task × Age, suggesting that the RPE was stable over time in both young and older participants. Thus, although overall memory performance was lower in older adults (which is compatible with previous research, see Hedden and Gabrieli, 2004; Nyberg et al., 2012), their RPE, did not show more deterioration over the span of the retention interval.

As expected, even stimuli that were novel during RP benefited from retrieval and were better recognized on Day 1 and Day 7 (hypothesis 5). This is compatible with previous work (Chan et al., 2006; Cho et al., 2017; Herweg et al., 2018; Guran et al., 2019) and further suggests that encountering stimuli in a retrieval mode leads to stronger memory traces irrespective of the stimulus Novelty. This cannot easily be explained by the Episodic Context Account or the fast-route to consolidation hypothesis. Instead, it might be explained by differences in difficulty and semantic elaboration: accordingly, retrieval is more effortful than encoding (Pyc and Rawson, 2009; Rowland, 2014), which may lead to deeper information processing as seen by slower response times (Phase 2) and activity increases in brain regions involved in long-term memory, such as the mesolimbic system (van den Broek et al., 2013; Herweg et al., 2018). Interestingly, *post hoc* analyses of the trend-level three-way interaction between Task, stimulus Novelty and Age groups suggest that the difference in the RPE for new, but not old, stimuli was more pronounced for young as compared to older adults (Figure 5). This may relate to age-related functional or anatomical changes of the underlying neural processes involved. Specifically, novelty processing relies on the dopaminergic system (Lisman and Grace, 2005; Lisman et al., 2011; Bunzeck et al., 2014), including the SN/VTA and MTL, which degenerates with age (Bäckman et al., 2006; Bunzeck et al., 2007; Düzel et al., 2010).

Importantly, RP had a stronger effect on recollection than familiarity rates (Figure 4, hypothesis 3). These findings further support the interpretation that RP leads to more semantic elaboration of practiced information in comparison to non-practiced information (Carpenter, 2009, 2011), which in turn increases recollection rates (Chan and McDermott, 2007). Indeed, previous studies could show a decrease of alpha and beta oscillations during RP (Guran et al., 2019), or stronger RP for less semantically bound word pairs (Carpenter, 2011), which further suggests a role of semantic processes or semantic elaboration in RP. However, in our study, familiarity rates were also significantly enhanced through RP, indicating that semantic elaboration is not the sole contributor to the RPE (Lehman et al., 2014).

The differential effect of RP on recollection and familiarity is compatible with dual process models of recognition memory and functional imaging studies (Yonelinas et al., 1996, 2010; Diana et al., 2007). They suggest different regions of the MTL to be involved in the two different memory experiences: the hippocampus and posterior parahippocampal gyrus are closely associated with recollection, the anterior parahippocampal gyrus, however, with familiarity (Diana et al., 2007). Therefore, the hippocampus appears to be more critical for recollection but not for familiarity (Yonelinas et al., 2010). Whether the effect of RP on recollection and familiarity is also mediated via these brain regions, needs to be investigated in future studies with lesion approaches or functional brain imaging techniques such as fMRI.

Based on a link between the dopaminergic system, Novelty Seeking, and declarative long-term memory (Benjamin et al., 1996; Ebstein et al., 1996; Schott et al., 2006; Krebs et al., 2009; Chowdhury et al., 2012), we expected a relationship between Novelty Seeking and RPEs (hypothesis 6). However, our results offer no convincing evidence that Novelty Seeking is linked to an individual’s benefit from RP. There was a marginal trend for the Novelty Seeking Scale “Exploratory Excitability” to be weakly positively linked to RP benefit. Future studies of the RPE should consider adding the TCI, or other tools to measure Novelty Seeking, to their paradigm. Alternatively, data across studies might be pooled to further increase sample size and power for this apparently small effect, and neuroimaging studies could directly investigate the hypothesized link between RP, Novelty Seeking, and dopaminergic neuromodulation.

In addition to the hypothesized effects, we observed significant interactions between Memory and stimulus Novelty, Memory and Day, as well as Day and stimulus Novelty. Regarding the interaction of Memory and stimulus Novelty, *post hoc* analyses indicates significant differences between old and new items only for recollection but not familiarity judgments. This suggests that stimuli that had been encountered more often had more opportunities to elicit elaborate associations that could later be recalled. *Post hoc* analysis of the significant interaction between Memory and Day revealed that recollection and familiarity scores were higher on Day 1 as compared to Day 7, but the difference between both days was more pronounced for recollection. In other words, higher recollection memory scores on Day 1 had a sharper decrease (i.e., higher forgetting rate) over time. The feeling of familiarity, which is supposed to be rather immediate and less resource intensive (Yonelinas, 2002; Basile and Hampton, 2013), was lower as compared to recollection (main effect Memory) but remained more stable from Day 1 to Day 7. From a more general perspective, the interaction of Memory and Day further underlines dual-process models mentioned above. And lastly, the interaction of Day and stimulus Novelty was driven by a more pronounced forgetting rate over time for new as compared to old stimuli. This effect can also be explained based on the different numbers of encounters. While new stimuli were presented only once, old stimuli were presented four times, which makes new stimuli more prone to forgetting.

Together, retrieval of information drives declarative memory at an immediate and longer retention interval. RP affects both types of recognition memory, yet the RPE is more pronounced for recollection as compared to familiarity. The RPE is pervasive in the sense that it is visible across the life span, yet older adults benefit less from retrieval as compared to younger adults. As such, our data give novel insights into the temporal stability of the RPE, differential effects of RP on recognition memory types, and they provide further evidence for age-related changes and decreases in the RPE.

## Data Availability Statement

The datasets generated for this study are available on request to the corresponding authors.

## Ethics Statement

The studies involving human participants were reviewed and approved by Ethikkommission der Universität zu Lübeck Ratzeburger Allee 160, Haus 2, 23538 Lübeck. The patients/participants provided their written informed consent to participate in this study.

## Author Contributions

C-NG designed the experiment, analyzed the data, and wrote the manuscript. JL-G collected the data and proofread the manuscript. NB supervised design and data-analysis, and wrote the manuscript.

## Conflict of Interest

The authors declare that the research was conducted in the absence of any commercial or financial relationships that could be construed as a potential conflict of interest.
